# Reemergence of Oropouche Virus in the Americas and Risk for Spread in the United States and Its Territories, 2024

**DOI:** 10.3201/eid3011.241220

**Published:** 2024-11

**Authors:** Sarah Anne J. Guagliardo, C. Roxanne Connelly, Shelby Lyons, Stacey W. Martin, Rebekah Sutter, Holly R. Hughes, Aaron C. Brault, Amy J. Lambert, Carolyn V. Gould, J. Erin Staples

**Affiliations:** Centers for Disease Control and Prevention, Fort Collins, Colorado, USA

**Keywords:** Oropouche virus, viruses, vector-borne infections, preparedness, response, United States

## Abstract

Oropouche virus has recently caused outbreaks in South America and the Caribbean, expanding into areas to which the virus was previously not endemic. This geographic range expansion, in conjunction with the identification of vertical transmission and reports of deaths, has raised concerns about the broader threat this virus represents to the Americas. We review information on Oropouche virus, factors influencing its spread, transmission risk in the United States, and current status of public health response tools. On the basis of available data, the risk for sustained local transmission in the continental United States is considered low because of differences in vector ecology and in human–vector interactions when compared with Oropouche virus–endemic areas. However, more information is needed about the drivers for the current outbreak to clarify the risk for further expansion of this virus. Timely detection and control of this emerging pathogen should be prioritized to mitigate disease burden and stop its spread.

Oropouche virus (genus *Orthobunyavirus*, Simbu serogroup) has recently been identified as a reemerging cause of widespread disease throughout the Americas (*1*). First discovered in Trinidad and Tobago in 1955, the virus caused periodic outbreaks of acute febrile illness in a limited number of countries in South and Central America for decades (*2*). Starting in late 2023, outbreaks of Oropouche virus disease were reported in areas with known endemic disease, and the virus emerged in new areas where it had not been historically documented. During January 1–September 6, 2024, more than 9,000 confirmed Oropouche virus disease cases and 2 deaths were reported from 6 countries: Bolivia, Brazil, Colombia, Cuba, the Dominican Republic, and Peru (*1*). In addition, several travel-associated cases have been reported among persons in the United States, Canada, and Europe traveling back from Cuba and Brazil (*1*,*3*,*4*). The recent expansion of the virus into previously nonendemic areas, identification of vertical transmission, and first reports of death from Oropouche virus disease have raised concerns about the broader threat this virus represents to the Americas, including the United States (*1*).

Oropouche virus circulates in both a sylvatic and an urban cycle. Sylvatic transmission, although not well understood, suggests a wide range of possible mammalian and avian hosts; virus has been detected in sloths (*Bradypus tridactylus*) and in several species of nonhuman primates, and antibodies have been found in domestic and wild birds and a rodent (*5*–*7*). Vectors hypothesized to be involved in sylvatic transmission include *Aedes serratus* and *Coquillettidia venezuelensis* mosquitoes (*2*,*8*). Humans develop sufficient viremia to contribute to viral spread, serving as bridge hosts that introduce Oropouche virus from its sylvatic maintenance cycle to populated areas. Once in the urban cycle, the virus circulates between humans and biting midges, *Culicoides paraensis* (*9*,*10*). The ubiquitous southern house mosquito, *Culex quinquefasciatus*, has also been suggested to play a role in urban transmission, although vector competency evaluations have shown mixed results (*11*–*13*) (Table; Figure 1).

**Table:** Possible vectors of Oropouche virus found in the United States and summary of laboratory and field data

Species	Laboratory evidence	Field data
*Culicoides paraensis* biting midge	Experimental infection from human to hamster through *Cu. paraensis* biting midge (*9*); efficient vector in laboratory studies (*9*)	Viral isolation from field collections during outbreaks in Para state, Brazil, 1978 (*14*); abundance correlated with higher seroprevalence in Para state, Brazil, 1975 (*10*)
*Culex quinquefasciatus* mosquito	Experimental infection from hamster to hamster via *Cx. quinquefasciatus* mosquito (*12*); found to be inefficient vector (possibly due to midgut barrier) in 1 study (*13*) but was found to have a low level of efficiency in other studies (*15**,**16*)	Viral Isolation from field collections in Para state, Brazil, 1961 and 1968 (*10*)
*Culicoides sonorensis* biting midge‡	Efficient vector in laboratory studies (*15**,**16*))	No viral isolations from field

**Figure 1:**
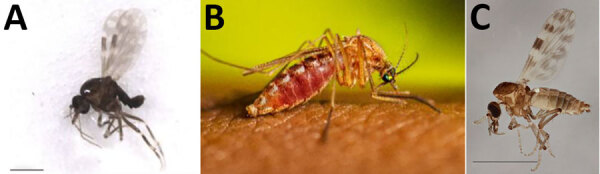
Possible biting midge and mosquito vectors of Oropouche virus found in United States in study of reemergence of Oropouche virus in the Americas, 2024. Possible vectors are presented in order of evidence for involvement in Oropouche virus transmission. A) *Culicoides paraensis* biting midge. Photo credit: “NACER355-12 Lateral”—BOLD:ABX5601 (compare *Culicoides paraensis*). Licensed under Creative Commons Attribution 4.0 International (https://creativecommons.org/licenses/by/4.0). B) *Culex quinquefasciatus* mosquito. Photo credit: Centers for Disease Control and Prevention Public Health Image Library. C) *Culicoides sonorensis* biting midge. Photo credit: Dominic Rose.

## Epidemiology and Clinical Manifestations

Outbreaks of Oropouche virus have affected both urban and rural areas, and attack rates can be high; ≈30% of the population can be infected (*17*). Sex-specific attack rates have been inconsistent; some outbreaks disproportionately affect female persons and others affect more male persons (*17*,*18*). Some studies have shown that younger persons are more likely to be infected, possibly because of lack of previous exposure and immunity to the virus (*17*). A recent analysis of >5,000 confirmed cases identified in January 2015–March 2024 in Brazil showed approximately equal proportions of confirmed cases among male and female persons, and most reported infections occurred in persons 20–49 years of age (*19*). Those data suggest that persons with different demographic traits can be infected with Oropouche virus and that infection is driven by exposures, which might vary by sex, age, and daily activity (*1*8).

The incubation period for Oropouche virus disease ranges from 3 to 10 days, and ≈60% of infected persons experience symptoms (*8*,*20*,*21*). Symptoms are similar to those of other vectorborne diseases, such as dengue, Zika, and chikungunya, and include acute onset of fever and severe headache, often with chills, myalgia, arthralgia, and fatigue. Other signs and symptoms can include photophobia, dizziness, retroorbital pain, nausea, vomiting, diarrhea, abdominal pain, conjunctival injection, and maculopapular rash (*17*,*22*,*23*). After the initial illness, up to 70% of persons can report relapse of symptoms, typically within a few days to weeks (*6*). Secondary episodes are clinically similar to the primary episode. No vaccines to prevent or medicines to treat Oropouche virus disease exist.

Although Oropouche virus disease is typically mild and reported deaths are rare, a small proportion of persons can develop more severe disease with hemorrhagic signs and symptoms (e.g., gingival bleeding, melena, and menorrhagia) or neurologic symptoms consistent with meningitis, meningoencephalitis, or Guillain-Barré syndrome (*1*,*21*,*23*,*24*). Of the 2 recent deaths associated with Oropouche virus among previously healthy young adult women, at least 1 patient had signs of hemorrhage (nasal, gingival, and vaginal bleeding and petechiae) starting 4 days after initial symptom onset (*1*). Neurologic symptoms have been reported in up to 4% of persons seeking clinical care (*25*). Signs and symptoms of neurologic disease can include occipital pain, dizziness, limb weakness, paresthesia, confusion, lethargy, photophobia, nausea, vomiting, nuchal rigidity, nystagmus, and paralysis (*17*,*24*,*25*). 

In June 2024, vertical transmission of Oropouche virus was identified when RNA was detected in a stillborn infant born to a pregnant woman who had symptoms of Oropouche virus disease at 30 weeks’ gestation (*1*). After this identification, a retrospective investigation identified 4 infants with microcephaly in whom Oropouche virus IgM was detected in serum samples or cerebrospinal fluid (CSF) samples collected shortly after birth (*26*). In August 2024, an additional infant with microcephaly associated with Oropouche virus infection was reported. The infant, born in June 2024, tested positive for Oropouche virus IgM on the second day of life in serum and CSF. The infant later died at 47 days of life, and multiple tissues tested positive for Oropouche viral RNA (*26*). Further investigation is required to determine the frequency of vertical transmission and whether the timing of Oropouche virus disease during pregnancy increases the risk for an adverse outcome.

## Testing

Testing for evidence of recent Oropouche virus infection can be performed on several different specimen types, though serum and CSF are used most often (*27*). During the first 7 days after infection, viral RNA can be detected through molecular testing such as reverse transcription PCR (RT-PCR). Most assays target the small (S) segment of the genome and cannot differentiate between Oropouche virus and other reassortant viruses (e.g., Iquitos virus) (*27*,*28*). After the first week of infection, antibody testing (e.g., IgM ELISA or plaque reduction neutralization test) is typically performed (*29*).

Viral RNA can be detected in the CSF of patients with neuroinvasive disease; however, it may not be present in the CSF at the time of clinical manifestation (because the virus is often cleared by that time), so serologic testing should be performed (*25*). Serologic testing is recommended for anyone experiencing a relapse of the disease because viral replication has not been detected during recurrence (*8*). Finally, in the event of fetal or infant death, postmortem tissues can be tested for evidence of antigen or viral RNA to assess causality (*1*).

## Factors Affecting Risk for Spread

The current outbreak in Latin America could be the result of lack of population-level immunity and viral reemergence in endemic areas, but other factors are possibly contributing to the spread and higher case counts. For example, changes to the viral genome through reassortment or vector distribution and competence might have resulted in more efficient transmission. Increased contact between humans and vectors caused by land use changes also could be contributing, because transmission activity has previously been detected in areas affected by deforestation (*2*). Finally, poor case recognition in the context of a large dengue outbreak could have furthered unchecked spread (i.e., because of lack of public health action when authorities are unaware of ongoing transmission).

Oropouche virus, like other orthobunyaviruses, is susceptible to reassortment, owing to its tripartite RNA genome, which includes the S segment encoding the nucleocapsid, medium (M) segment encoding the glycoproteins, and large (L) segment encoding L protein, which has RNA-directed RNA polymerase functions (*30*,*31*). The strain causing the current outbreak has shown some evidence of successive reassortment with genetically similar viruses (e.g., Perdões virus, Iquitos virus). Although the manner in which this strain might have influenced vector competence, disease severity, virus transmissibility, and immune protective status is not clear, preliminary research suggests reduced cross-neutralization with prototype strains in vitro (*32*). Reassortment has been observed with other orthobunyaviruses in the Americas (e.g., Fort Sherman virus, Potosi virus) and experimentally between Oropouche virus and orthobunyaviruses in the Simbu serogroup from outside the Americas (*30*,*31*,*33*).

Limited data exist regarding the specific vectors associated with recent urban outbreaks, although viral RNA has historically been detected in biting midges, including *Cu. paraensis*, and in *Cx. quinquefasciatus* mosquitoes (*10*,*14*). *Cu. paraensis* midges are found throughout the tropics, subtropics, and temperate areas in the Americas in wetland, forest, agricultural, rural, and periurban areas. In addition, *Cx. quinquefasciatus* mosquitoes are relatively ubiquitous, having a broad distribution in the northern and southern hemispheres. Temporally, outbreaks in Latin America have mostly coincided with the rainy season, during which biting midge and mosquito populations are typically more abundant (*17*,*34*).

Currently, large dengue outbreaks are occurring throughout the world; the Americas have reported unprecedented numbers of cases totaling >11 million since late 2023 (*35*). Because Oropouche virus disease and dengue have similar symptomology, they are difficult to distinguish clinically, and dengue testing is usually conducted before Oropouche virus testing is considered (*29*). This factor, combined with limited Oropouche testing availability, could have led to an underrecognition of increasing disease burden, which in turn might have led to a further expansion of outbreak and spread of the virus through infected persons into new areas.

## Risk for Sustained Local Transmission of Oropouche Virus in the United States

As of September 2024, local transmission of Oropouche virus had not been reported in the United States, although some cases have been reported in travelers (*4*; https://www.cdc.gov/oropouche/data-maps.) Various factors are likely to affect the risk for local spread of the virus, including the rate of introduction from travel-associated cases, the presence and distribution of the vectors and potential host reservoirs, and potential virus adaptation.

Recent experiences with the introduction of chikungunya and Zika viruses to the United States could foretell what might occur with Oropouche virus, because all 3 arboviruses are maintained in an urban cycle between humans and arthropod vectors. During the chikungunya outbreak in 2014–2015, ≈3,700 travel-associated cases were reported in the continental United States. Despite thousands of possible introductions of viremic travelers, only 13 locally transmitted cases were identified in very limited areas of Florida and Texas (*36*). During the Zika virus outbreak in 2016–2017, US jurisdictions reported 5,389 travel-associated cases, resulting in 231 locally acquired cases, which also occurred in limited areas of Florida and Texas (*37*). Sustained local transmission of chikungunya and Zika was successfully thwarted by vector control and other public health interventions. Those experiences suggest that, even with frequent virus introductions through infected persons into the continental United States, large urban outbreaks of Oropouche are unlikely. For US territories, 4,900 locally acquired chikungunya cases were reported during 2014–2015 and 37,052 locally acquired cases of Zika virus were reported during 2016–2017 (*36*,*37*). Most of those cases were reported from Puerto Rico. On the basis of available data, the risk for sustained local transmission in the continental United States is likely low, whereas the risk for sustained transmission in Puerto Rico and the US Virgin Islands is unknown.

Most travel-associated Oropouche cases detected in Europe and the United States have been in travelers from Cuba (*3*,*4*). Cuba is in midst of its peak rainy season, which is associated with increased vector abundance (*17*,*34*), suggesting that more travel-associated cases might be expected from there. Previous research has not reported the primary vector of *Cu. paraensis* biting midges in Cuba, although *Cx. quinquefasciatus* mosquitoes and several biting midges of the *Ceratopogonidae* family have been detected there, including *Cu.* *furens* biting midges, which are also present in Florida (*38*). Vector competency evaluations have not been completed for many of those species, and a better understanding of transmission ecology in the Cuba outbreak and in the Dominican Republic will help to assess risk to the United States and, in particular, Puerto Rico.

**Figure 2:**
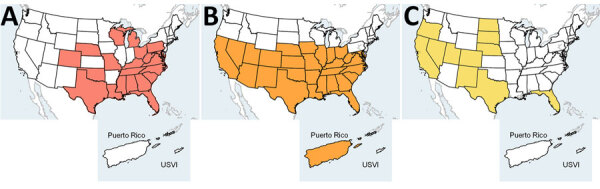
Distribution of biting midge and mosquito vectors in the United States and select territories based on field observations and modelling in study of reemergence of Oropouche virus in the Americas, 2024. Possible vectors are presented in order of evidence for involvement in Oropouche virus transmission. A) *Culicoides paraensis* biting midge; B) *Culex quinquefasciatus* mosquito; C) *Culicoides sonorensis* biting midge. Presence of vectors in a jurisdiction does not imply uniform distribution throughout an entire geographic area. A zone exists where *Cx. quinquefasciatus* mosquitoes hybridize with other *Culex* species; this zone is not accounted for in the map because no vector competence studies for Oropouche virus for those species have been conducted. USVI, US Virgin Islands.

Both chikungunya and Zika viruses in the United States are transmitted by *Aedes (Stegomyia)* mosquitoes, which oviposit and develop in containers in and around homes, making persons more susceptible to mosquito exposure and, ultimately, infection. In contrast, the primary Oropouche vector, the *Cu. paraensis* biting midge*,* has low abundance in North America and mostly resides in tree holes in the southeast and midwestern United States (*39*–*41*) (Figure 2). In addition, the *Cu. sonorensis* biting midge is an another possible Oropouche vector, according to laboratory competency evaluations (*15*,*16*). Located mainly west of the Mississippi, this biting midge would be unlikely to perpetuate local Oropouche virus transmission in humans, because it is found in rural areas around livestock operations (*15*,*42*). Overall, taken together, the spatial distribution of biting midges in rural areas and poor vector competence in laboratory studies of mosquitoes translate to reduced risk for urban transmission in the United States, if *Cu. paraensis* biting midges are indeed the primary vector in ongoing Oropouche outbreaks.

Finally, despite its extreme abundance and enormous geographic range, the *Cx. quinquefasciatus* mosquito is not a very competent vector in laboratory studies and is the target of extensive West Nile virus vector control efforts (*15*,*43*,*44*). Existing control programs could therefore be adapted to the Oropouche context. On the other hand, *Cx. quinquefasciatus* mosquitoes have demonstrated widespread resistance to pyrethroids (particularly in parts of Florida), which could blunt the efficacy of vector control efforts. *Cx. quinquefasciatus* mosquitoes could represent a more serious threat to increase the risk for local transmission if it proves to be a competent vector. Of note, many mosquito (and *Culicoides* midge) species in the United States, which feed primarily on humans, have not been tested for vector competence of Oropouche virus.

Sylvatic transmission of Zika and chikungunya viruses has only been documented in Africa and relies on mosquitoes and nonhuman primates, whereas Oropouche virus maintenance in sylvatic settings can rely on wide array of species, on the basis of viral isolation and detection of antibodies in many different species (*6*). Oropouche virus has not been isolated or detected in birds, but Oropouche virus antibodies have been identified in >11 different families of wild and domestic birds in Brazil, raising questions about their role in transmission (5,*14*). Should the virus infect wild bird populations in North America, it is possible that Oropouche virus could become endemic, similar to the progression for West Nile virus. Oropouche virus’s propensity for reassortment could affect its ability to infect new hosts, enhance vector competence, and evade host immune response (*45*). However, the probability of sustained local transmission at this time is thought to be low in the continental United States because Oropouche virus would be required to overcome a series of biologic and ecologic obstacles.

## Preparedness for and Response to Oropouche Virus in the United States

In the past 25 years, the United States has experienced and responded to 4 different emergent mosquitoborne viral diseases, caused by West Nile, chikungunya, Zika, and dengue viruses. Given those experiences, preparation for potential Oropouche virus introductions into the United States could rely on several existing tools and interventions, including the current public health surveillance systems, case identification, vector control, personal protection, and public health communication.

ArboNET, the US national arboviral surveillance system, was established in 2000 in response to West Nile virus and can be adapted to capture data about new emerging and reemerging arboviruses (https://www.cdc.gov/oropouche/data-maps/current-year-data.html). ArboNET enables reporting of human disease cases, human infections (e.g., presumptive viremic donors), animal disease, sentinel animal infections, and vector infections. Human disease cases are reported from state and territorial health departments using standard case definitions. Case reports can include information on travel location, clinical manifestations, and transmission mechanisms (*46*).

Oropouche virus disease is not a nationally notifiable condition, but state and territorial health departments can voluntarily report identified cases to ArboNET. In addition, if Oropouche virus emerges in the United States, the Council of State and Territorial Epidemiologists can decide whether to make Oropouche virus disease nationally notifiable and determine whether a new case definition should be developed to capture potential fetal deaths or congenital infections, as was done for Zika virus previously (*47*).

Clinicians should report suspected Oropouche virus disease cases to state or local health departments to enable testing and to implement community prevention measures and messaging. Information about clinical features, diagnosis, and clinical management is available on the Centers for Disease Control and Prevention (CDC) website (https://www.cdc.gov/oropouche/hcp/clinical-overview). At this time, testing for Oropouche virus should be considered when a patient has traveled within 2 weeks of initial symptom onset (because patients can experience recurrent symptoms) to an area with documented or suspected Oropouche virus circulation and has an abrupt onset of fever, headache, and >1 of the following signs/symptoms: myalgia, arthralgia, photophobia, retroorbital/eye pain, or indications of neuroinvasive disease (e.g., stiff neck, altered mental status, seizures, limb weakness, or cerebrospinal fluid pleocytosis). If concern exists for local transmission in a nonendemic area, providers should consider whether the patient had contact with a person with confirmed Oropouche virus infection, lives in an area where travel-related cases have been identified, or has known vector exposure (e.g., mosquitoes or biting midges). In addition, testing should only be considered among patients who tested negative for other pathogens, in particular dengue. If strong suspicion of Oropouche virus disease exists on the basis of the patient’s clinical features and history of travel to an area with virus circulation, providers should not wait on negative test results before sending specimens to CDC. This guidance on clinical case identification will likely need to be modified as the epidemiologic situation evolves, including whether local transmission is identified, and as more is learned about clinical manifestations and transmission risk, including for vertical transmission and potential adverse birth outcomes.

Available vector control tools, such as insecticide spraying and source reduction (modification of larval habitats to prevent oviposition), are similar for biting midges and mosquitoes, but questions remain about the application of such tools in the context of Oropouche. Empirical evaluations of *Cu. paraensis* midge–specific control measures are lacking. Previous works have shown aerial spraying of the insecticide naled has resulted in substantial reduction (up to 99%) in pestiferous *Culicoides* species (*48*). Source reduction around dairy operations for *Cu. sonorensis* midges and removal of leaf waste for *Cu. paraensis* midges have also been used, with varying degrees of success (*49*). *Cx. quinquefasciatus* mosquitoes are abundant and widely distributed; therefore, control activities should be determined on the basis of mosquito surveillance data to more efficiently target when and where this species is active. A combination of larviciding and adulticiding will be most useful, given the asynchronous hatching of this mosquito’s egg rafts. Challenges in implementing vector control include the limited scope of vector control agencies that primarily target mosquitoes and are not mandated to manage other arthropods, lack of acceptability of aerial spraying of insecticides, insecticide resistance, and limited utility of larval source reduction because of the cryptic nature of some larval habitats (i.e., tree holes for *Cu. paraensis* midges) (*39*).

Persons can protect themselves against both midge and mosquito bites by wearing long sleeves and pants and by using an insect repellant registered by the US Environmental Protection Agency. Those products are safe for pregnant and breastfeeding women when used as directed; for children <3 years of age, products containing oil of lemon eucalyptus or para-menthane-diol should not be used. Windows and door screens can also prevent mosquitoes from entering the home and protect against vectorborne diseases (*50*). However, *Culicoides* spp. midges are smaller than typical window screen holes and can pass through and enter the home. Mesh size 20 (or 20 × 20 mesh, which has 20 openings in 1 linear inch) is designed to exclude biting midges. Patients with suspected Oropouche virus disease should avoid being bitten by biting midges and mosquitoes for 1 week to prevent infection of naive vectors.

Strong engagement with the community is necessary to gain support for vector control activities, as well as to improve the uptake of personal protective measures, which are currently the only ways to prevent infection. Rapidly distributing information to public health professionals, providers, the public, and other stakeholders can lead to improved surveillance, diagnosis, and implementation of prevention strategies. The use of various platforms for distribution of communications (e.g., CDC website, Health Alert Network messages, social media posts, publications, and data dashboards) can improve the reach and distribution of messages about Oropouche virus and ways persons can prevent themselves from being infected and spreading the virus.

## Summary

Overall, on the basis of current knowledge, the risk for localized outbreaks of Oropouche virus disease in most areas in the United States should be considered low because of differences in vectors and human-vector interactions (e.g., mitigation by widespread availability of closeable windows and air-conditioning) compared with endemic areas. Some states and territories are probably at elevated risk for local spread, including those where infected travelers are most likely to arrive and be readily exposed to vectors, such as southern Florida or Puerto Rico. Past experiences with several emerging and reemerging vectorborne diseases, as well as new information from Oropouche outbreaks (e.g., transmission ecology in Cuba), will help to inform and refine preparedness, detection, and response to Oropouche virus. Public health partners should prioritize timely detection and control of this emerging pathogen to prevent human disease cases and the spread of the virus.
